# The Effectiveness of Ultrasound Visual Biofeedback in Articulation Therapy for Children and Adolescents With Speech Sound Disorders: A Systematic Review and Meta‐Analysis

**DOI:** 10.1111/1460-6984.70209

**Published:** 2026-02-18

**Authors:** Oi Yan Yiu, Wilfred Hing Sang Wong, Felicia Pokta, Agnes Yun Ting Fong, Winnie Wan Yee Tso, Patrick Ip

**Affiliations:** ^1^ Department of Paediatrics and Adolescent Medicine The University of Hong Kong Hong Kong China; ^2^ Department of Allied Health Hong Kong Children's Hospital Hong Kong China; ^3^ Services for the Elderly, Caritas Hong Kong Hong Kong China; ^4^ Department of Otorhinolaryngology, Head and Neck Surgery The Chinese University of Hong Kong Hong Kong China

**Keywords:** articulation accuracy, meta‐analysis, paediatric speech therapy, speech sound disorders, systematic review, ultrasound visual biofeedback

## Abstract

**Background:**

Speech sound disorders (SSD) may compromise speech intelligibility in children and adolescents, impacting communication, social interactions and academic performance. Ultrasound visual biofeedback (U‐VBF) has emerged as a promising tool for articulation therapy, providing real‐time tongue movement visualization, yet its evidence base requires updating given methodological advancements.

**Aims:**

This systematic review and meta‐analysis updates the evidence for the effectiveness of the use of U‐VBF in improving articulation accuracy for paediatric SSD, synthesizing recent studies with robust designs to quantify outcomes and inform clinical adoption.

**Methods:**

Following PRISMA guidelines, a preregistered review (PROSPERO: CRD42024627408) involved searching PubMed, Web of Science, CINAHL Plus, Cochrane Library and Linguistics and Language Behaviour Abstracts (January 2000–July 2025) for studies on U‐VBF in children and adolescents (≤18 years) with SSD, using all designs with articulation accuracy outcomes. Three reviewers used Covidence for study selection, data extraction and risk of bias assessment. Busk and Serlin's d2 effect sizes at the study level were calculated where possible for random‐effects meta‐analysis.

**Main Contribution:**

From 545 references, 35 studies (192 unique participants, aged 4–18) were included (16 for meta‐analysis). The pooled d2 was 1.32 (95% CI [0.54, 2.10]), indicating substantial improvements in accuracy despite high heterogeneity (*Q* = 494.36, *p* < 0.001; *I* ^2^ = 97.0%) and variability (range –0.26 to 4.78). SSD subtype‐specific patterns and intensity influences emerged, but small samples limited subgroup analyses. Funnel asymmetry (*z* = 2.478, *p* = 0.013) suggested potential publication bias. Implications include enhanced intensity reporting for personalized protocols, though generalizability is restricted by factors including the predominance of English‐speaking participants.

**Conclusions:**

Evidence indicates that U‐VBF is promising for SSD articulation therapy, supported by stronger designs post‐2019. Future RCTs, non‐English studies and standardized reporting are essential to mitigate bias and refine individualized applications.

**WHAT THIS PAPER ADDS:**

*What is already known on this subject*

- A 2019 systematic review qualitatively synthesized early evidence for ultrasound visual biofeedback (U‐VBF) in speech sound disorders (SSDs), highlighting its promise as an adjunct for persistent errors but limited by small samples, few RCTs and no effect size pooling. Subsequent studies have introduced more robust designs and quantitative data, necessitating an update to quantify U‐VBF's impact amid growing clinical interest.

*What this paper adds to existing knowledge*

- This review updates the evidence with 35 studies (192 unique participants) and a novel meta‐analysis of 16 studies, yielding a large pooled effect on articulation accuracy despite high heterogeneity. It identifies SSD subtype‐specific variability and intensity influences, revealing reporting gaps that limit subgroup analyses. These findings provide the first quantitative benchmark, enabling future power calculations and stratified research.

*What are the potential or actual clinical implications of this work?*

- U‐VBF offers a viable tool for personalized SSD therapy, with enhanced intensity reporting guiding tailored protocols. Clinicians should integrate U‐VBF judiciously, mindful of English‐centric evidence and potential bias inflating effects. Future research should prioritize RCTs and non‐English trials to refine applicability across diverse populations.

## 1 Introduction

Speech sound disorders (SSD) may severely compromise one's ability to produce intelligible speech, which are associated with limitations in communication, social interactions and academic performance (McCormack et al. 2011, 2009, Foster et al. 2023). Socioemotional difficulties have been reported in school children with speech errors, with greater impact observed in older children (Hitchcock et al. 2015; Wren et al. 2023). With SSD forming a substantial part of speech therapy caseloads globally, surveys report that although speech‐language pathologists (SLPs) acknowledge the potential efficacy of instrumental methods such as ultrasound and electropalatography (EPG), their application remains minimal (McLeod and Baker 2014). Recent studies reveal growing interest in technology‐supported tools, despite concerns about over‐reliance on device screens and potential distractions from non‐therapy app features (Furlong et al. 2021), clinicians have expressed increasingly favourable attitudes toward ultrasound biofeedback, notwithstanding ongoing accessibility barriers (Dugan et al. 2023). In light of this shifting landscape, the use of ultrasound visual biofeedback (U‐VBF) has become an attractive tool that helps clients and clinicians overcome these hurdles by providing real‐time visual feedback on tongue movement and supplementary data, enabling precise cueing and adjustment and presenting a new paradigm for augmenting therapy in children with SSD.

U‐VBF constitutes an evolution in speech therapy for the management of articulation, building upon earlier visual biofeedback methods such as EPG. EPG is effective in tracking tongue–palate contact during speech production but requires custom‐fitted palates, limiting accessibility. In contrast, advancements in ultrasound technology have introduced systems with fast frame rates which are more accessible (Cleland et al. 2022), providing dynamic images of tongue movements which allow clinicians and children to visualize and adjust articulatory patterns in real time. Both techniques have unique merits—EPG excels in precise contact data, while U‐VBF facilitates broader tongue shape observation without custom hardware—yet the lower cost and ease of use of U‐VBF have contributed to its growing prominence in clinical and research settings. This is particularly valuable for paediatric populations, where clinical observations suggest that children may find abstract verbal cues challenging to implement, due to their developmental stage. There is increasing evidence supporting the use and effectiveness of U‐VBF in achieving improved speech outcomes across various subtypes of SSDs (Sugden et al. 2019). As the use of U‐VBF becomes increasingly available, the potential of this approach to transform clinical practice continues to grow, warranting a comprehensive synthesis of the latest evidence.

The need to update the evidence base for U‐VBF stems from considerable advancements since the foundational systematic review by Sugden et al. (2019) was published. That review collated early studies on U‐VBF for developmental SSD, which indicated its potential to be an effective adjunct for persistent speech errors. However, the evidence was limited by small sample sizes, the largest being 13 and predominantly lower‐strength designs, including only one randomized controlled trial (RCT). Its findings were further limited by the lack of effect size reporting in the included studies. Since that time, more recent studies have reported RCTs with larger sample sizes (McAllister et al. 2020, Preston et al. 2020, Cleland et al. 2022, McCabe et al. 2023) that have advanced the evidence base with more robust designs, while other studies (e.g. Cleland et al. 2019, Benway et al. 2021, Spencer et al. 2023) have provided data allowing proper calculation of effect size for meta‐analysis, further strengthening the quantitative evidence base through statistical power.

This systematic review and meta‐analysis aims to update the evidence for U‐VBF in paediatric SSD, extending the work of the published review by combining data from recent studies with robust designs and to evaluate effectiveness using pooled effect sizes of articulation accuracy outcomes. The findings are intended to equip SLPs with evidence‐based insights to inform the adoption of U‐VBF in clinical practice and direct future research toward individualized interventions for children and adolescents with SSD that enhance cross‐study comparability and build a more robust evidence base.

## 2 Methods

### 2.1 Protocol Registration

The systematic review was conducted in accordance with established methodological guidelines (Higgins et al. 2019) and reported following the Preferred Reporting Items for Systematic Reviews and Meta‐Analyses (PRISMA) (Page et al. 2021). It was registered on the international systematic review registry PROSPERO (CRD42024627408).

### 2.2 Eligibility Criteria

Studies were included based on the following PICO criteria: Population—children and adolescents aged under 18 with SSD; Intervention—U‐VBF aimed at articulation; Comparator—speech or articulation therapy (as an active comparison condition) or a no‐treatment control group; Outcome—articulation accuracy (e.g. percentage of consonants correct [PCC], speech intelligibility or patient‐reported speech outcomes). All study designs were eligible, including RCTs, cohort studies, case‐control studies and single‐case studies. Studies were excluded if they were not treatment studies, involved adults or reported outcomes unrelated to speech sound production.

### 2.3 Search Strategy

A systematic literature search was conducted using the following databases: PubMed, Web of Science, CINAHL Plus, Cochrane Library and Linguistics and Language Behaviour Abstracts, from January 2000 to July 2025. Compared to Sugden et al. (2019), this protocol adopts a post‐2000 cutoff to focus on contemporary ultrasound advancements and excludes studies with adult participants for a stricter paediatric emphasis, while expanding database coverage. Search terms targeted four concepts: ultrasound, speech therapy, speech disorders and child. Medical Subject Headings (MeSH) were used in PubMed search, for example, ‘ultrasonography’, ‘speech therapy’, ‘speech disorders’ and ‘child’, with keyword variations adapted for other databases, with Boolean operators (AND) to combine terms. No language restrictions were applied. The full search strategy is provided in Table S1 in supplementary material.

### 2.4 Study Selection and Data Extraction

Study selection and data extraction were conducted using Covidence, an online systematic review tool. Three SLPs participated as reviewers; two independently screened titles and abstracts, reviewed full texts to determine inclusion and extracted data from eligible articles. Discrepancies were resolved by consensus during videoconferences. Extracted data included study characteristics (e.g. country of origin, study design), participant details (e.g. demographics, medical background), intervention specifics (e.g. dose, dose frequency, total intervention duration) and speech outcome measures (e.g. PCC, time point of assessment). For meta‐analysis, Busk and Serlin's d2 effect sizes, Cohen's d, Hedges’ g or data enabling their calculation (e.g. means of pre‐ and post‐treatment data, standard deviations) were extracted, to evaluate articulation accuracy outcomes.

### 2.5 Risk of Bias Assessment

Risk of bias was evaluated using standard tools within Covidence. Two reviewers independently rated each study and reached a consensus when discrepancies arose. For non‐RCTs, the Risk of Bias in N‐of‐1 Trials (RoBiNT) Scale (Tate et al. 2013) was employed, comprising 15 items related to internal and external validity (e.g. design, blinding, treatment adherence, data analysis). These items are rated as 0, 1 or 2 points where higher scores reflect lower bias, with a maximum of 30 points. For RCTs, the Risk of Bias 2 (RoB 2) tool (Sterne et al. 2019) was used to assess five domains: randomization process, deviations from intended interventions, missing outcome data, measurement of the outcome and selection of reported result, yielding judgments of low risk of bias, some concerns or high risk of bias. Bias ratings informed meta‐analysis sensitivity analyses, which test primary result robustness by excluding high risk studies (defined as RoBiNT scores <10/30 or RoB 2 ‘high risk of bias’).

### 2.6 Data Synthesis and Meta‐Analysis

To prepare for meta‐analysis, Busk and Serlin's d2 effect size, Cohen's d or Hedges’ g were extracted directly from included studies if available. For studies that reported sufficient data (e.g. means and standard deviations at pre‐ and immediate post‐treatment, as other follow‐up time points varied widely across studies and lacked consistency for pooled analysis), individual‐level d2 (for single‐case experimental designs [SCEDs]) or Cohen's d/ Hedges’ g (for groups) were calculated based on study type (e.g. d2 as the mean difference divided by the pooled standard deviation; Busk and Serlin (1992)). For multi‐participant studies, individual d2s were then aggregated to study‐level d2 via weighted summarization (inverse variance using standard errors [SEs], approximated using proxies such as the reported number of trials in probe lists where SEs were unreported (Borenstein et al. 2009)). Busk and Serlin's d2 was adopted to report the effect size of SCEDs, while Cohen's d or Hedges’ g was used for group studies. Studies of case study/case series, or with insufficient data for deriving effect size would not be included in meta‐analysis.

A random‐effects meta‐analysis was conducted for eligible studies, prioritizing d2 for SCEDs, with narrative synthesis for group studies where pooling was infeasible due to limited data. The DerSimonian‐Laird method was used to pool effect sizes in Jeffrey's Amazing Statistics Program (JASP), given the anticipated heterogeneity in study designs and populations (DerSimonian and Laird 2015). This model accounts for both within‐study sampling error and between‐study variability by assuming that the true effect size differs across studies due to factors such as population differences or methodological variations, rather than a single fixed effect, providing a more conservative estimate suitable for heterogeneous data. Heterogeneity was assessed using Cochran's Q test. Sensitivity analyses excluded studies with high risk of bias—defined as scores below 10 points on RoBINT or high risk of bias on RoB 2, to test robustness of results by evaluating changes in heterogeneity (Q), effect size and predictive interval. Forest plot showing effect sizes and confidence intervals for individual studies alongside the pooled overall effect, and funnel plot of effect sizes against study precision measure to detect asymmetry indicative of publication bias, were generated in JASP.

## 3 Results

**FIGURE 1:**
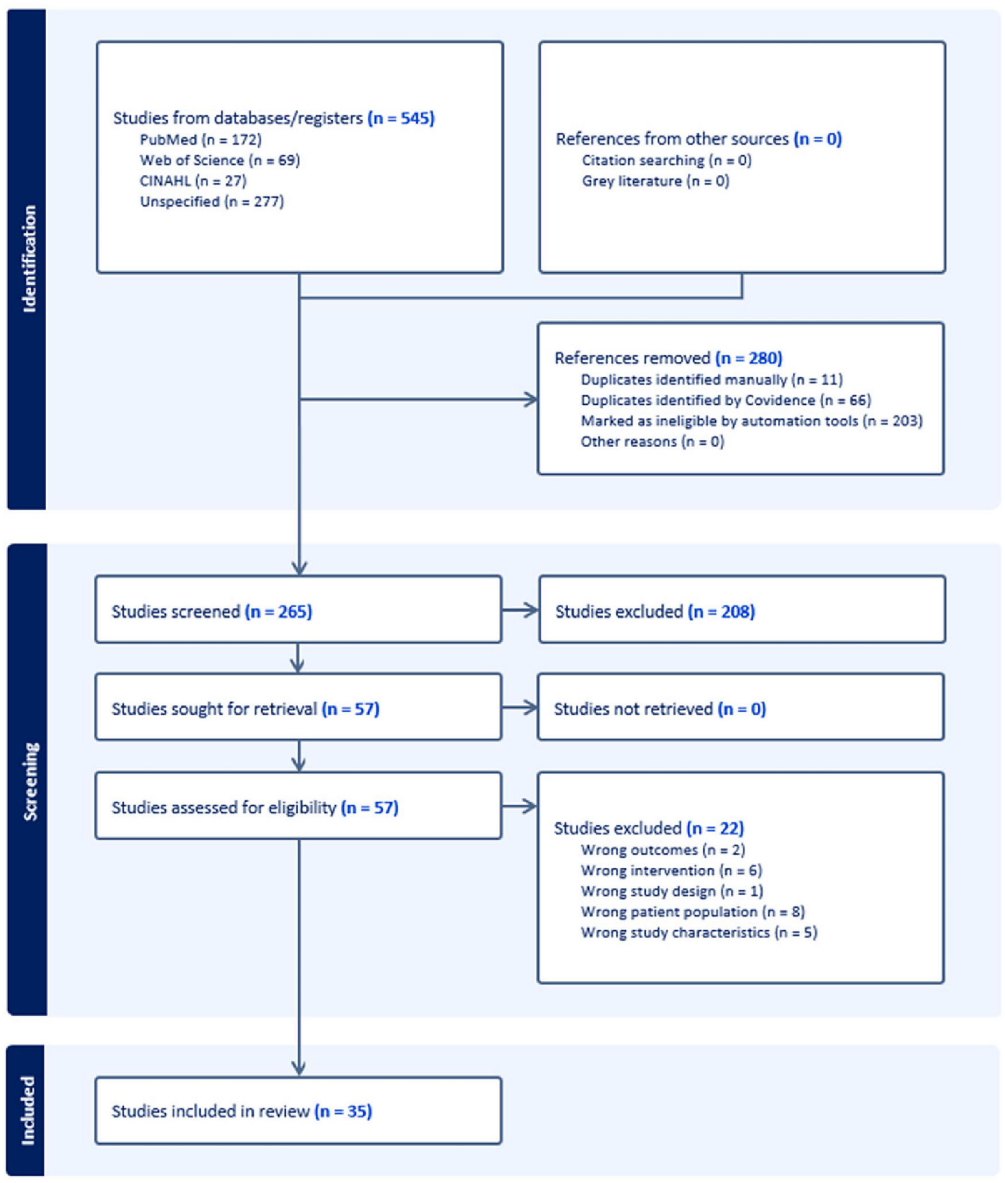
Preferred reporting items for systematic reviews and meta‐analyses flowchart of the process of study selection.

The study selection process for this systematic review is illustrated in a PRISMA flow chart (Figure 1). From 545 references imported, 77 duplicates were removed and 203 were excluded as irrelevant by automated screening tools, leaving 265 for title and abstract review. Of the 265 studies screened by title and abstract, 208 were excluded. Fifty‐seven studies underwent full‐text review, with 22 excluded for reasons stated in the chart. Thirty‐five studies, reported in 34 articles (including one article reporting two studies), were included for analysis.

### 3.1 Study Characteristics

The 35 studies spanned four countries: the USA (*n* = 20, 57.1%), Canada (*n* = 9, 25.7%), the UK (*n* = 4, 11.4%) and Australia (*n* = 2, 5.7%). This distribution highlights a significant Anglophone bias, as English is the official or primary national language in all included countries. These studies included 13 case studies and case series, 16 SCEDs and six group designs. Among the 16 SCEDs, 11 were of multiple baseline design (68.8%). Other SCED types included alternating treatment designs with randomization (*n* = 1, 6.3%), randomized block design (*n* = 1, 6.3%), alternating treatment designs (*n* = 2, 12.5%), as well as a two‐phase design (*n* = 1, 6.3%). The six group designs (17.1%) consisted of RCT (*n* = 4, 66.7%) and two quasi‐experimental designs of single‐group pretest‐posttest design (*n* = 1, 16.7%) and a control group design (*n* = 1, 16.7%).

### 3.2 Participant Characteristics

The studies enrolled a total of 234 participants, including 17 in control groups who did not receive U‐VBF therapy. After excluding controls, accounting for 13 participants who appeared in multiple included studies (Bernhardt et al. 2005, Bacsfalvi et al. 2007, Cleland and Scobbie 2021, Raaz et al. 2021) and removing 12 who withdrew or were lost to follow‐up (including participants from McCabe et al. (2023) and Preston et al. (2024)), 192 unique individuals who received U‐VBF therapy were included in this review. Their ages ranged from 4 to 18 years, with the majority aged 6–16 years (see Table 1 for details). Gender, specific SSD details and comorbid diagnoses were not fully reported across all studies. The most identified SSD subtypes included childhood apraxia of speech (CAS) and residual speech sound disorders. Other reported comorbidities potentially related to SSD encompassed hearing loss, cleft palate, auditory processing disorders and 22q deletion syndrome. Most studies (*n* = 28, 80.0%) indicated that their participants had received prior speech therapy. Excluding the duplicated participants mentioned earlier, two individuals had previously undergone speech therapy with ultrasound techniques (Bacsfalvi 2010, Hitchcock and Byun 2015). Some authors described the prior speech therapy as traditional without biofeedback techniques, while others did not explicitly report on this aspect. Details of participants’ gender and description are reported in Table S2 in supplementary material.

**TABLE 1:** Study, participant and treatment details of the included studies.

Study (Country)	Study design	Participants	Treatment details	
		*N*, age, gender	Approaches, target	Intensity
Bernhardt et al. 2003 (Canada)	Single‐group pre‐post case series	*n* = 4, 16;0‐18;0, 3M, 1F	U‐VBF, EPG /s/, /ʃ/, /ɹ/, /l/, /i/, /ɪ/, /u/, /ʊ/	*Dose*: Trials not reported, 30‐min session *Dose frequency*: 1 time/week *Total intervention duration*: 14 weeks (14 sessions)
Bernhardt et al. 2005 (Canada)	Individual case study design	*n* = 4, 16;0‐18;0, 3M, 1F	U‐VBF, EPG /s/, /ʃ/, /ɹ/, /l/, /i/, /ɪ/, /u/, /ʊ/	*Dose*: Trials not reported, 30‐min session *Dose frequency*: 1 time/week *Total intervention duration*: 14 weeks (14 sessions)
Adler‐Bock et al. 2007 (Canada)	Individual case study design	*n* = 2, 12;0‐14;0, 2M	U‐VBF with traditional therapy /r/	*Dose*: Trials not reported, 60‐min session *Dose frequency*: Not reported *Total intervention duration*: 6–20 weeks (13 sessions)
Bacsfalvi et al. 2007 (Canada)	Single‐group pre‐post case series	*n* = 3, 18;0, 2M, 1F	U‐VBF, EPG Feedback Therapy /i/, /ɪ/, /u/, /ʊ/	*Dose*: Trials not reported, 45‐min or 60 to 90‐min session *Dose frequency*: 2 times/week *Total intervention duration*: 6 weeks (12 sessions)
Bernhardt et al. 2008 (Canada)	Case series (BCB research design)	*n* = 13, 7;0‐15;0, 8M, 5F	U‐VBF consultation, traditional treatment /ɹ/, sibilants	*Dose*: Not reported *Dose frequency*: Not reported *Total intervention duration*: Not reported (14‐19 sessions)
Modha et al. 2008 (Canada)	SCED—Alternating treatment design	*n* = 1, 13;0, 1F	U‐VBF, traditional therapy /ɹ/	Dose: Trials not reported, 30–45 min session Dose frequency: 1 time/week Total intervention duration: 9 weeks (9 sessions)
Bacsfalvi 2010 (Canada)	SCED—Multiple baseline design	*n* = 3, 15;0‐18;0, 1M, 2F	U‐VBF /r/	*Dose*: Trials not reported, 45‐min session *Dose frequency*: 1 time/ week *Total intervention duration*: 7–8 weeks (7–8 sessions)
Lipetz and Bernhardt 2013 (Canada)	SCED—Single‐subject two‐phase case study	*n* = 1, 15;9, 1M	U‐VBF with traditional therapy hierarchies, U‐VBF with palatrography and voice training /s/, /z/, /ʃ/, /ʧ/	*Dosage*: Trials not reported, 60‐min session *Dosage frequency*: 6 sessions over 8 weeks, 5 sessions over 4 weeks *Total intervention duration*: 22 weeks (11 sessions—Phase I: 6 sessions, Break: 10 weeks, Phase II: 5 sessions)
Preston et al. 2013 (USA)	SCED—Multiple baseline design	*n* = 6, 9;0‐15;0, 6M	U‐VBF with traditional approaches sound sequences	*Dose*: 228 trials (average), 60‐min session *Dose frequency*: 2 times/week *Total intervention duration*: 10–16 weeks (18 sessions)
Byun et al. 2014—Study 1 (USA)	SCED—Multiple baseline design	*n* = 4, 6;1‐10;3, 2M, 2F	U‐VBF rhotics	*Dose*: 60 treatment trials, 30–45 min session *Dose frequency*: 2 times/week *Total intervention duration*: 8 weeks (16 sessions)
Byun et al. 2014—Study 2 (USA)	SCED—Multiple baseline design	*n* = 4, 7;8‐15;8, 2M, 2F	U‐VBF rhotics	*Dose*: 60 treatment trials, 30–45 min session *Dose frequency*: 2 times/week *Total intervention duration*: 8.5 weeks (17 sessions)
Cleland et al. 2015 (UK)	Case series	*n* = 7, 6;0‐10;1, 5M, 2F	U‐VBF with articulation hierarchy or motor‐based approach /k/, /g/, /r/, /ʃ/, /t/	*Dose*: Trials not reported, 60‐min session *Dose frequency*: 1 time/week *Total intervention duration*: 12 weeks (12 sessions)
Hitchcock and Byun 2015 (USA)	Single‐case study design	*n* = 1, 11;02, 1F	U‐VBF guided by CPF /r/	*Dose*: 60 trials, 30–45 min session *Dose frequency*: 1 time/week *Total intervention duration*: 11 weeks (11 sessions)
Lee et al. 2015 (USA)	Case study	*n* = 1, 13;0, 1M	U‐VBF /r/	*Dose*: Trials not reported, 30‐min session *Dose frequency*: 1 time/week *Total intervention duration*: 12 weeks (12 sessions)
Bressmann et al. 2016 (Canada)	Randomized controlled trial	*n* = 6, 7;0‐10;0, 5M, 1F	U‐VBF, articulation therapy /ɹ/	*Dose*: Trials not reported, 60‐min session *Dose frequency*: 1 time/week *Total intervention duration*: 10 weeks (10 sessions)
Heng et al. 2016 (Australia)	SCED—Multiple baseline design	*n* = 2, 4;0‐4;11, 1M, 1F	U‐VBF /k/, /g/	*Dose*: 59.3 trials (mean in pre‐practice), 61.7 trials (mean in practice), 50‐min session *Dose frequency*: 2 times/ week *Total intervention duration*: 3 weeks (6 sessions)
Preston et al. 2016a (USA)	Case series	*n* = 3, 10;8‐14;3, 3M	U‐VBF with chaining, auditory perception training /r/, /s/, /tʃ/	*Dose*: Trials not reported, 60‐min session *Dose frequency*: 1–2 times/day *Total intervention duration*: over 10 days (16 sessions)
Preston et al. 2016b (USA)	SCED—Multiple baseline design	*n* = 3, 10;0‐13;0, 3M	U‐VBF /ɹ/	*Dose*: 142 trials (mean), 60‐min session *Dose frequency*: Not reported *Total intervention duration*: Not reported (14 sessions)
Roxburgh et al. 2016 (UK)	Single‐case study design	*n* = 2, 6;2‐9;2, 2M	U‐VBF with articulation hierarchy or motor‐based approach /n/, velars	*Dose*: Trials not reported, 60‐min session *Dose frequency*: 1 time/ week *Total intervention duration*: 16 weeks (16 sessions)
Sjolie et al. 2016 (USA)	SCED—Single‐subject randomized block design	*n* = 4, 7;0‐9;7, not reported	U‐VBF with chaining procedures /ɹ/	*Dose*: 215 trials (average), 51–55 min session *Dose frequency*: 2 times/ week *Total intervention duration*: 7–8 weeks (14 sessions)
Preston et al. 2017a (USA)	SCED—Multiple baseline design	*n* = 12, 10;1‐16;7, 8M, 4F	U‐VBF with principles of motor learning (PML) /ɹ/	*Dose*: PML + U‐VBF 287.4 trials (average); PML + No‐U‐VBF 300.4 trials (average), 60‐min session *Dose frequency*: 2 times/ week *Total intervention duration*: 7 weeks (14 sessions)
Preston et al. 2017b (USA)	SCED—Alternating‐treatments, single‐subject experimental design	*n* = 6, 8;2‐16;8, 4M, 2F	U‐VBF, Auditory Perception Training /ɹ/, /s/	*Dose*: 69 practice trials in PROS, 98 practice trials in Non‐PROS (average), 60‐min session *Dose frequency*: 2 times/week *Total intervention duration*: 7 weeks (14 sessions)
Preston et al. 2018 (USA)	SCED—Multiple baseline design	*n* = 12, 8;2‐16;10, 12M	U‐VBF guided by CPF Vocalic /ɹ/	*Dose*: up to 162 practice attempts, 50‐min session *Dose frequency*: 2 times/week *Total intervention duration*: 8 weeks (16 sessions)
Cleland et al. 2019 (UK)	Case series	*n* = 15, 6;1‐13;4, 12M, 3F	U‐VBF with motor‐based therapy approach /k/, /ɛ/, /ʃ/, /s/	*Dose*: Around 100 attempts, 60‐min session *Dose frequency*: 1 times/week *Total intervention duration*: 10–12 weeks (10 sessions)
Preston et al. 2019 (USA)	SCED—Multiple baseline design	*n* = 12, 8;11‐14;3, 9M, 3F	U‐VBF guided by CPF. /ɹ/	*Dose*: 139 practice trials in UVF‐enhanced treatment; 133 practice trials in traditional treatment (mean), 50‐min session *Dose frequency*: 2 times/week *Total intervention duration*: 8 weeks (16 sessions)
Preston et al. 2020 (USA)	Randomized controlled trial	Randomized: *n* = 38, 8;0‐16;0, 23M, 15F Analyzed: n = 36, not reported	U‐VBF guided by CPP. /ɹ/	*Dose*: 155.8 practice trials in U‐VBF; 141.9 practice trials in P+U‐VBF (mean), 60‐min session *Dose frequency*: 2 times/week *Total intervention duration*: over 7 weeks (14 sessions)
Benway et al. 2021 (USA)	SCED—Single‐case randomization design	*n* = 7, 9;5‐15;8, 3M, 4F	U‐VBF with visual‐acoustic biofeedback, traditional visual and auditory placement cues /ɹ/	*Dose*: Up to 200 practice trials, 101‐min session *Dose frequency*: 2 times/week *Total intervention duration*: 5 weeks (10 sessions)
Cleland and Scobbie 2021 (UK)	Single‐group pre‐post case series	*n* = 5, 6;1‐13;2, 3M, 2F	U‐VBF with traditional visual and auditory placement cues /k/	*Dose*: Trials not reported, 60‐min session *Dose frequency*: 1 time/week *Total intervention duration*: ∼10 weeks (ranged from 9 to 12 sessions)
Gibson and Lee 2021 (USA)	SCED—Single‐subject experimental, multiple baseline design	*n* = 2, 4;7‐5;11, 2F	U‐VBF, Motor‐phonetic articulation approach /k/, /g/	*Dose*: 40 trials (average), 30‐min session *Dose frequency*: 2 times/week *Total intervention duration*: over 9–10 weeks (17‐19 sessions)
Raaz et al. 2021 (USA)	Case report	*n* = 1, 9;0, 1M	U‐VBF guided by SAILS and CPP /ɹ/	*Dose*: 162 trials, 60‐min session *Dose frequency*: 2 times/week *Total intervention duration*: 7 weeks (14 sessions)
McAllister et al. 2022 (USA)	Single‐group pretest‐posttest design	*n* = 33, 9;0‐15;0, not reported	U‐VBF with traditional treatment guided by CPP /ɹ/	*Dose*: Phase I & IIa—Up to 24 correct productions + 96 trials as structured practice. Phase IIb—Up to 24 correct productions + 216 trials as structured practice, 45–90 min session *Dose frequency*: 2–3 times/week *Total intervention duration*: 10 weeks (22 sessions)
McCabe et al. 2023 (Australia)	Randomized controlled trial	Enrolled: *n* = 14, 6;5‐13;0, 11M, 3F Analysed: *n* = 13, not reported	U‐VBF with motor chaining procedures, ReST. Not reported	*Dose*: 185.2 trials in U‐VBF and 99.8 trials in ReST (mean), 55‐60‐min session *Dose frequency*: 2 times/ week *Total intervention duration*: 6 weeks (12 sessions)
Spencer et al. 2023 (USA)	Control group study	Tx group: *n* = 16, 8;0‐12;6, 10 M, 6F TD group: *n* = 17, 8;5–13;7, 9M, 8F	U‐VBF guided by CPF /ɹ/	*Dose*: up to 162 trials, 45‐min session *Dose frequency*: 2 times/ week *Total intervention duration*: 8 weeks (16 sessions)
Preston et al. 2024 (USA)	Randomized controlled trial	Enrolled: *n* = 56, 9;1‐16;8, 39M, 17F Analysed: *n* = 45, not reported	U‐VBF with motor chaining Not reported	*Dose*: 230 trials in distributed U‐VBF; 248 trials in distributed No‐U‐VBF; 238 trials in distributed telepractice; 438 trials in intensive + U‐VBF; 386 trials in intensive + No‐U‐VBF 336 trials; 212 trials in intensive + telepractice 212 trials, (average), 60‐min session *Dose frequency*: Intesntive ‐ 10 times/week for week 1, 3 times/week for week 2–3, 2 times/week for week 4–5. Distributed ‐ 2 times/ week *Total intervention duration*: 5 or 10 weeks (20 h)
Hashemi Hosseinabad and Xing 2024 (USA)	SCED—Single‐subject multiple baseline experiment	*n* = 5, 6;5‐13;5, 2M, 3F	U‐VBF with motor‐based therapy /r/, /s/, /sh/	*Dose*: 150–350 attempts per target segment, 45‐min session *Dose frequency*: 2 times/week *Total intervention duration*: 8 weeks (16 sessions)

*Note*: M = Male, F = Female, U‐VBF = Ultrasound‐Visual Biofeedback, EPG = Electropalatography. CPF = Challenge Point Framework, PROS = Prosodic, CPP = Challenge Point Program, ReST = Rapid Sayllable Transitions.

### 3.3 Intervention Details

Participants received interventions targeting sounds that were either individualized or consistent within studies (e.g. English /ɹ/ across all participants). Approaches predominantly featured U‐VBF, often combined with or alternated with methods such as traditional therapy (*n* = 8, 22.9%), motor‐based approaches (*n* = 8, 22.9%; e.g. phonetic placement or dynamic cueing techniques emphasizing articulatory movement, which often overlap with traditional therapy but were reported distinctly in studies), EPG (*n* = 3, 8.6%), auditory perception training (*n* = 3, 8.6%), with conditions ranging from sequential use to combined or optional applications of U‐VBF. Regarding treatment intensity, most studies (*n* = 33, 94.3%) reported session durations, which ranged from 30 to 101 min, though exact trial doses were not always specified. More recent studies, however, tended to document trial doses (*n* = 23, 65.7%), reporting targeted attempts or the average number of productions achieved within a session or across the entire treatment period, ranging from 50 to 300 attempts per session. Dose frequency varied widely, from three times per month to an intensive schedule of 10 times per week, with 1–2 times per week being the most common (*n* = 28, 80.0%). The shortest intervention duration was 10 days, while the longest lasted 22 weeks. Interventions were primarily delivered by SLPs or supervised SLP trainees (e.g. graduate students). Details on treatment targets, approaches and intensity are reported in Table 1. Some common components in treatment included pre‐practice or elicitation (e.g. initial modelling and cueing to elicit target sounds before structured practice; *n* = 12, 34.3%), structured practice (*n* = 13, 37.1%) and the use of hierarchical approaches, such as linguistic levels (*n* = 11, 31.4%) and the challenge point framework (*n* = 6, 17.1%). Additional details on personnel, conditions and session components are provided in Table S2 in supplementary material.

### 3.4 Outcome Measures and Treatment Effect

All 35 studies incorporated perceptual evaluations of speech production, with outcomes detailed in Table 2. The majority (82.9%) assessed target sound accuracy across treated and untreated words or consonants, utilizing word probes across linguistic levels at different time points, enabling some studies to report treatment, generalization and maintenance effect. Other than binary choice in rating sound accuracy, some studies adopted measures including speech acceptability judgments, perceptual ratings on the degree of ‘on‐target’, transcription ratings, collectively indicating any enhanced speech production aligned with target sounds. A relatively lower percentage of studies (5.7%) utilized PCC measure and the Intelligibility in Context Scale (ICS) to assess overall speech accuracy and how understandable speech is to listeners. Treatment effects across the 35 included studies were predominantly positive, with improved speech accuracy and intelligibility reported in 26 studies (74.3%). These included substantial gains in target sound production (e.g. target sound accuracy increases of 30.0%–95.0%), generalization to untreated items and maintenance at follow‐up. However, the remaining nine studies (25.7%) showed mixed or limited results, such as variable participant responses or non‐sustained effects. These patterns varied by design, with positive effects observed in 69.2% of case studies/case series (9/13) and 66.7% of group studies (4/6), compared to 81.3% (13/16) for SCEDs—whose pooled effect is addressed via meta‐analysis later.

**TABLE 2:** Outcomes, quality rating and meta‐analysis eligibility of the included studies.

Study	Outcomes		Quality rating	Meta‐Analysis
				eligible (Y/N)
	Outcome measures	Treatment effect		
Bernhardt et al. 2003	TSA for treated and untreated consonants in single words	TSA improved significantly for treated (36%) versus untreated (15%) consonants across participants. No significant difference in effectiveness between EPG and ultrasound technologies.	10/30	N
Bernhardt et al. 2005	Perceptual ratings on consonants and vowels in nonsense word samples	Significantly improved ratings noted in some participants or sounds.	9/30	N
Adler‐Bock et al. 2007	TSA for targeted words at word and phrase levels	TSA increased at phrase level from pre‐ to post‐treatment for both participants.	9/30	N
Bacsfalvi et al. 2007	Transcription ratings for trained and untrained vowels in CVC words	Vowel production improved toward adult English targets, with changes in transcription ratings for some vowels.	7/30	N
Bernhardt et al. 2008	TSA for single words of standardized test containing target sounds	TSA improved in eleven participants. Greater gains were observed in participants having more consultation time with ultrasound.	8/30	N
Modha et al. 2008	TSA for targeted consonants in probes of various context, pre‐, mid‐, and post‐treatment	TSA improved throughout treatment, with or without ultrasound.	7/30	N
Bacsfalvi 2010	TSA for targeted consonants in word and syllable probes	Significant /r/ TSA improvement at word level observed in one participant only.	8/30	Y
Lipetz and Bernhardt 2013	TSA of target words in tasks at different linguistic level from single word to spontaneous narrative production	Similar production accuracy after Phase I, while improvement noted after Phase II and mostly maintained in 4‐months follow‐up time.	8/30	Y
Preston et al. 2013	TSA for treated and untreated sound sequences and PPC, before and within treatment and 2‐month follow‐up	All participants achieved 80% accuracy for 2 consecutive sessions for some treated sound sequences, maintained at 2 months. PPC increased significantly from pre‐treatment to follow‐up.	12/30	Y
Byun et al. 2014—Study 1	TSA for treated and untreated words with targeted sounds, baseline, treatment, and maintenance	Minimal treatment effects; half of participants showed no clinically meaningful effect size.	18/30	Y
Byun et al. 2014—Study 2	TSA for treated and untreated words with targeted sounds, baseline, treatment, and maintenance	Generalization to untreated words without biofeedback observed, with large to very large effect sizes for all participants.	18/30	Y
Cleland et al. 2015	TSA for untreated word probes, baseline, mid‐, and post‐therapy	TSA increased by 95% (range 70–100%) from baseline to 6 weeks post‐therapy. 100% non‐overlapping data across participants indicated high effectiveness.	13/30	N
Hitchcock and Byun 2015	TSA for /r/ in word and sentence probes, baseline, treatment, maintenance, and follow‐up	/r/ TSA reached ∼100% at word and sentence levels, maintained through follow‐up. Effect sizes ranged from ‐0.82 to 37.6 for vocalic/consonantal /r/.	16/30	N
Lee et al. 2015	TSA of target sound and non‐treatment target in one‐syllable word	Increase in production accuracy of targets across treatment and maintained for 2 weeks after treatment.	8/30	N
Bressmann et al. 2016	Speech acceptability judgments; sound classification (perceived as target or not)	Significant improvement in final and follow‐up assessments versus intake. No significant difference between biofeedback and non‐biofeedback groups.	Some concerns	N
Heng et al. 2016	TSA for treated and untreated sounds at syllable and word levels, acquisition, retention, and generalization	One participant showed acquisition and retention; no improvement in another participant.	10/30	Y
Preston et al. 2016a	TSA for targeted sounds in onset and rhyme positions	All participants showed acquisition (effect sizes 0.4–16.1); generalization and retention results were mixed across participants.	18/30	N
Preston et al. 2016b	TSA for treated and untreated words during treatment training	TSA increased in two participants during practice trials, but no generalization to untreated words observed.	14/30	Y
Roxburgh et al. 2016	Listener judgments (perceived as closer to target); TSA for targeted consonants	Significant TSA improvement for both participants in VAM and U‐VBF therapies from baseline to maintenance. One participant showed possible deterioration in U‐VBF.	12/30	N
Sjolie et al. 2016	TSA for targeted sounds in acquisition, retention, and generalization probes	Two participants showed acquisition; one had significant ultrasound advantage (d = 0.78). No significant retention or generalization differences between ultrasound and non‐ultrasound sessions.	18/30	Y
Preston et al. 2017a	TSA for generalization probes and sentence imitation, pre‐ and 2‐month follow‐up	TSA increased by 29.7% (PML+US) and 33.2% (PML+No US), with effect sizes of 4.9 and 6.16. Retention observed at 2‐month follow‐up (16% to 45%).	17/30	Y
Preston et al. 2017b	TSA for untrained word‐level generalization probes	TSA increased by 38.1% (prosodic) and 31.0% (non‐prosodic), with effect sizes of 14.52 and 8.31, respectively.	19/30	Y
Preston et al. 2018	TSA for untreated word probes, baseline, treatment, and maintenance	TSA improved across all participants (median effect size 6.16). High‐frequency (HF) UVF outperformed low‐frequency (LF) UVF; treatment order (HF‐LF vs. LF‐HF) impacted gains.	15/30	Y
Cleland et al. 2019	TSA for untreated wordlists; ICS	TSA showed variable generalization; five children had no effect, others had small to large effects. ICS increased significantly from baseline to maintenance in some children.	19/30	N
Preston et al. 2019	TSA for untreated word probes	U‐VBF outperformed traditional treatment (effect sizes ‐2.4 to 21.75 vs. ‐0.39 to 8.78). Early U‐VBF yielded better outcomes; individual responses varied.	17/30	Y
Preston et al. 2020	TSA for untrained word probes, pre‐, mid‐, post‐treatment, and 2‐month follow‐up	No significant difference between U‐VBF and P+U‐VBF groups; both showed 34% TSA increase, retained at 2 months.	Low risk of bias	N
Benway et al. 2021	TSA for untrained word probes	Three participants showed clinically significant generalization (effect sizes 1.71–3.11) to untreated words post‐treatment.	16/30	Y
Cleland and Scobbie 2021	TSA for treated word probes	All participants showed positive TSA changes from pre‐ to post‐intervention, with maintenance in three participants at 6 weeks post‐treatment.	14/30	N
Gibson and Lee 2021	TSA for treated and untreated sound probes, baseline, treatment, and post‐intervention	TSA reached 80% for target sounds, with maintenance at 2 weeks and generalization at 2 months post‐intervention.	12/30	Y
Raaz et al. 2021	TSA for trained and untrained liquid phoneme probes, baseline, mid‐, and post‐treatment	Perceptual accuracy for /ɹ/ and /l/ improved substantially, indicating generalization to untreated contexts.	13/30	N
McAllister et al. 2022	TSA for untreated word probes with target sounds	TSA increased by 38% on average; four participants showed clinically significant responses. Low‐stimulability cluster responded strongly to ultrasound; high‐stimulability cluster gained in both treatments.	16/30	N
McCabe et al. 2023	PPC and prosodic severity in untreated word and sentence probes, pre‐, post‐, and 1‐month follow‐up	Large PPC improvements from pre‐ to post‐treatment with ReST and U‐VBF, retained at 1 month. Prosodic severity also improved significantly.	Low risk of bias	N
Spencer et al. 2023	TSA for untreated word probes with target sounds	Participants showed varied responses to U‐VBF, with mean effect size of 5.25 indicating clinically significant generalization.	12/30	Y
Preston et al. 2024	TSA for untreated phrase probes at 5, 10, and 15 weeks	U‐VBF showed significant TSA gains at 5 weeks, not sustained at 10 weeks. Intensive treatment enhanced generalization at all time points.	Low risk of bias	N
Hashemi Hosseinabad and Xing 2024	TSA for untreated word probes, baseline, mid‐, post‐therapy, and maintenance; ICS	TSA increased significantly (effect sizes 1.11–3.17); generalization varied. ICS improved post‐treatment and remained stable during maintenance.	17/30	Y

*Note*: TSA = Target Sound Accuracy, EPG = Electropalatography, CVC = Consonant‐Vowel‐Consonant, PPC = Percentage Phoneme Correct, VAM = Visual Articulatory Model, PML = Principle of Motor Learning, US = Ultrasound, ICS = Intelligibility In Context, U‐VBF = Ultrasound‐Visual Biofeedback, ReST = Rapid Sayllable Transitions.

### 3.5 Quality Ratings

Quality evaluation of the 35 studies was performed via two methods, with each study's quality ratings presented in Table 2. Thirty‐one were rated with the RoBiNT on a 30‐point scale, with scores ranging between 7/30 and 19/30 (median: 13), suggesting moderate quality overall. External validity generally scored higher than internal validity, with studies commonly having deficits in study design, randomization and blinding. Conversely, four studies were assessed using RoB 2 because of their RCT design, with three being ‘low risk of bias’ and one ‘some concerns’. Higher scores or ratings indicated more robust designs, although variability across studies was apparent. Following completion of outcomes and quality evaluation, meta‐analysis was conducted.

### 3.6 Meta‐Analysis

Eligibility for meta‐analysis of each study is detailed in Table 2. Of the 35 studies, SCEDs and group studies were further assessed on their eligibility to be included in meta‐analysis. The majority of the SCEDs include calculable effect sizes (*n* = 15). Among the six group studies, only two of them provide adequate data for Cohen's d and Hedges’ g calculation (Bressmann et al. 2016, Preston et al. 2020). Another study (Spencer et al. 2023) reported individual d2 of each participant in one single group and is comparable to other SCEDs. No meta‐analysis was implemented for group studies due to the small sample size (*n* < 5). The 15 SCEDs and 1 group study with single‐group effect size reporting were put under meta‐analysis, with the detailed data extracted for calculation listed in Table S3 in Supplementary material.

**FIGURE 2:**
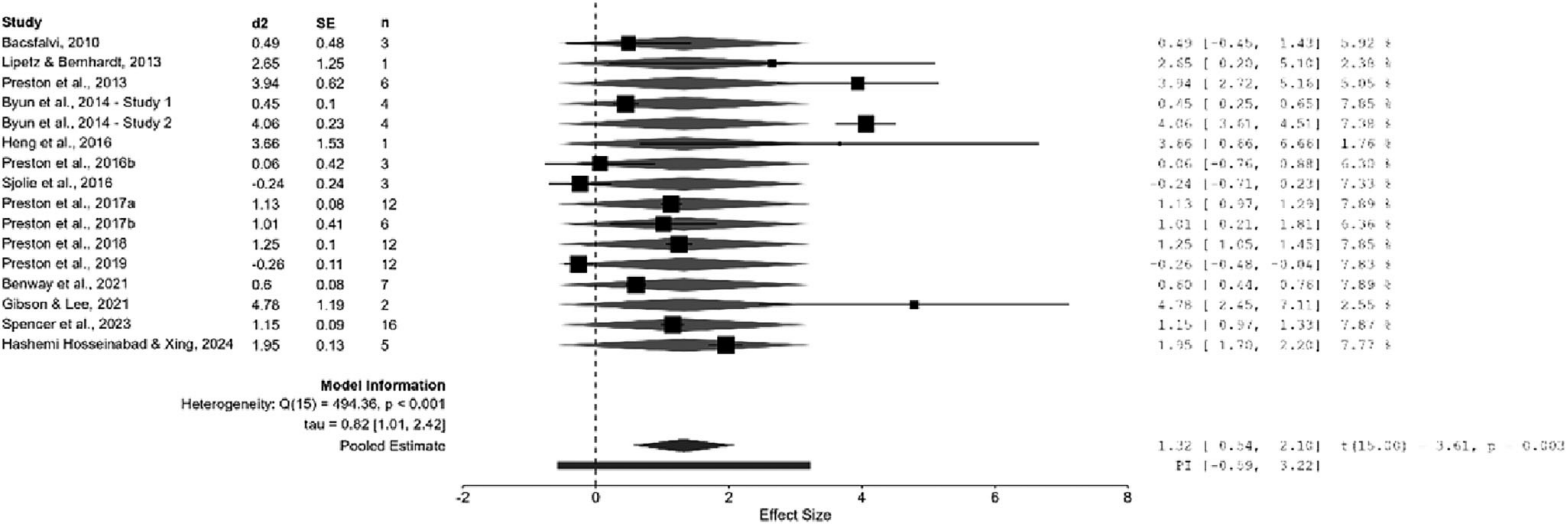
Forest plot of study‐specific and pooled d2 effect sizes (*n* = 16 Studies).

**FIGURE 3:**
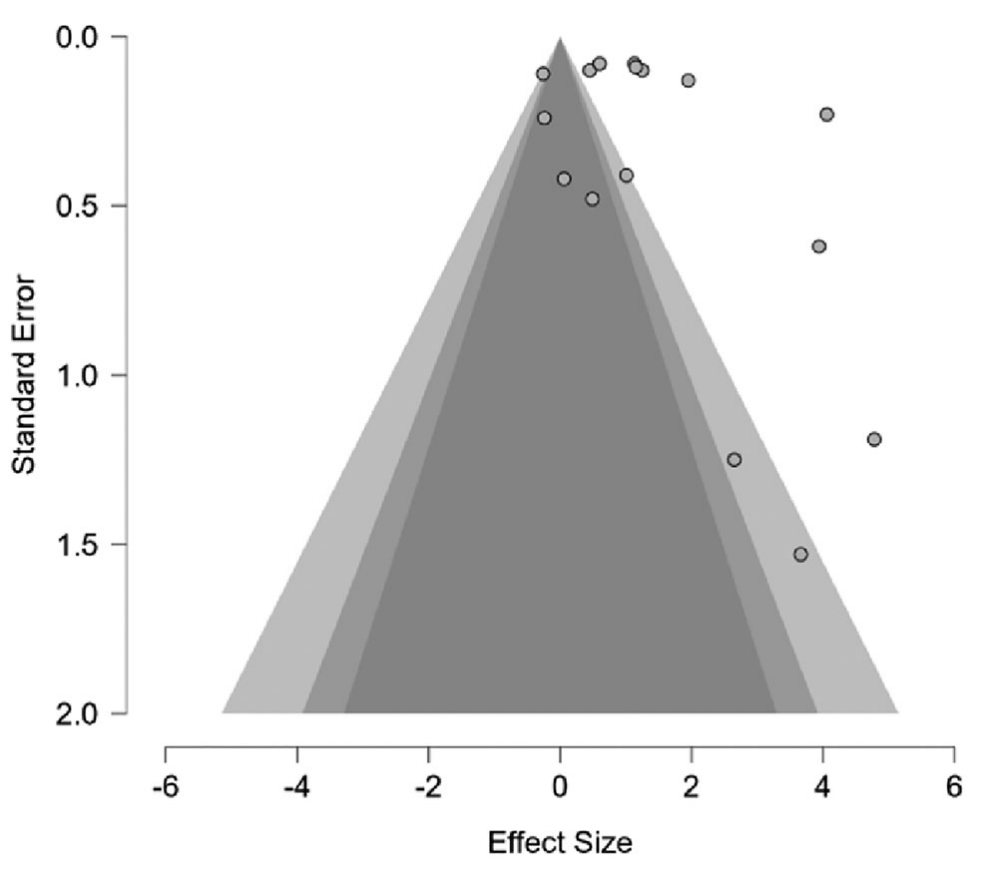
Funnel plot of study effect sizes (d2) against standard error.

Cochran's *Q* test revealed significant heterogeneity among the studies (*Q* = 494.36, *p* < 0.001), with *I* ^2^ = 97.0% indicating considerable between‐study variability (Higgins et al. 2003). The estimated between‐study variance (Tau^2^) was 0.665 [95% CI, 1.016, 5.877], which indicates moderate‐to‐high variability in true effect sizes across studies. Figure 2 shows the forest plot for speech accuracy outcomes, with most effect sizes positive, ranging from −0.26 to 4.78. The pooled effect size was 1.32 (95% CI [0.54, 2.10], *p* < 0.001). The predictive interval was −0.59 to 3.22. The funnel plot (Figure 3) showed rightward asymmetry, confirmed with the meta‐regression test (*z* = 2.478, *p* = 0.013), suggesting potential publication bias (Egger's test threshold: |*z*| > 1.96, Harrer et al. (2021)), consistent with patterns in small‐sample speech therapy reviews where null results from underpowered studies may be underrepresented. To further assess result robustness, two studies at high risk of bais indicated by RoBiNT scores less than 10/30 were excluded from the meta‐analysis for sensitivity analyses. These analyses revealed decreased but still significant heterogeneity (*Q* = 491.63, *p* < 0.001), a larger effect size and a predictive interval that continued to include zero. Therefore, all studies were retained in order to preserve statistical power with a larger sample.

## 4 Discussion

This systematic review extends the foundational work of Sugden et al. (2019) by updating the evidence for the effectiveness of U‐VBF for SSD in the paediatric population. Of an initial pool of 545 references, 35 studies were included (13 new since Sugden et al. (2019)), encompassing 192 unique participants aged 4–18 years, adding to the evidence base with a larger dataset focusing on the paediatric population. Furthermore, a quantitative synthesis via meta‐analysis of 16 studies—primarily SCEDs plus one single‐group reported in a control group study—was conducted to complement the prior review's qualitative approach. This broader dataset, incorporating stronger designs such as additional RCTs and more SCEDs eligible for meta‐analysis and a large pooled effect size in the quantitative synthesis, contributes to a more comprehensive assessment of U‐VBF's emerging position in research and clinical practice.

### 4.1 Developments in Evidence and Methodology Since 2019

Since Sugden et al. (2019)’s review, this study indicates improvement in the geographical representation, study designs and reporting practices that strengthen the quality of U‐VBF research for paediatric SSD. Geographical distribution is still biased toward North America, yet the UK and Australia have become emerging contributors. These findings may indicate a gradual diversification potentially due to wider access to ultrasound technology or research grants. This shift contrasts with the previous review's almost exclusive focus on North America, though the persistent Anglophone and WEIRD (Western, Educated, Industrialized, Rich, Democratic) skew represents a systemic limitation in the field, restricting insights into diverse linguistic typologies and professional contexts. This limited global representation highlights the urgent need for initiatives to support research in underrepresented regions such as Asia and broaden the evidence base beyond non‐English languages.

Study designs have diversified, with SCEDs still prevailing (16 of 35 studies, 45.7%), but the inclusion of five group designs comprising four RCTs represents a change from the scarcity of RCTs before 2019. This shift has enhanced reliability by minimizing bias and increased sample sizes, especially with half of the post‐2019 studies featuring cohorts larger than the maximum of 13 participants noted by Sugden et al. (2019), although the continued dominance of SCEDs restricts generalizability. Despite methodological maturity, quality ratings were similar, with the median RoBiNT score being 13 in this review compared to 12 in Sugden et al. (2019) in which challenges in randomization and blinding continue. Reporting has also improved, with 66.0% of studies now reporting dose (targeted or actual trials per session) versus 37.9% in Sugden et al. (2019), demonstrating better treatment intensity description in accordance with Warren et al. (2007)’s framework, which includes dose, dose form, dose frequency, total intervention duration and cumulative intervention intensity. Preliminary evidence from visual biofeedback specific reviews supports the value of this progress, revealing small but significant relationships between treatment intensity and outcomes (Hitchcock et al. 2019). This improvement, complemented by regular reporting of session durations (94.3%) and frequency (80.0% at 1–2 times/week), highlights a growing awareness of the role of intensity in therapy outcomes. These advances enable more accurate study replication, support the development of individualized interventions and provide a basis for comparing outcomes across studies according to various treatment intensities.

### 4.2 Treatment Effect and Its Implications

The meta‐analysis of eligible studies yielded a moderate‐to‐large pooled effect on speech accuracy in children and adolescents with SSD, but with wide variability in individual study effect sizes and a predictive interval crossing zero (Figure 2). This variability could be related to several influencing factors, such as participant factors (SSD subtype, comorbidities, age) and treatment intensity (dose, dose frequency and duration). Within studies on specific disorders such as CAS, effect sizes ranged widely from 0.24 to 2.65, illustrating intra‐group variability potentially due to participant age or prior therapy. Effect sizes of individual study also varied across disorders (e.g. 0.49 and 4.78 for hearing loss, 1.95 for cleft palate with various comorbidities, as compared to CAS just mentioned), suggesting subtype‐specific responsiveness to U‐VBF. Similarly, treatment intensity influenced outcomes: Two studies with comparable protocols (162 trials per session) yielded similar effect sizes (d2 = 1.15 and 1.25), while another with slightly reduced number of trials per session (∼149) and sessions yielded a much lower effect (d2 = −0.24), highlighting how multiple factors—including intensity, subtype and comorbidities—underlie the observed effect size variability. However, with only 16 studies in the meta‐analysis, formal subgroup analyses were not feasible, precluding confirmation of these hypotheses and underscoring the need for larger datasets. These patterns contribute to the overall heterogeneity observed.

The high heterogeneity and predictive interval are indicative of inconsistent effects among studies, a common phenomenon in meta‐analyses given variations in study design, sample size and intervention protocol. Heterogeneity related to study design may stem from discrepancies among SCEDs including sample size, as well as differences in intervention intensity (e.g. dose frequency and duration, reported variably across studies). In contrast, heterogeneity tied to individual differences may arise from participant variability, including SSD subtypes and comorbidities as discussed earlier, as well as their ages, different targeted speech sounds, levels of complexity or prior therapy exposure with or without U‐VBF.

The slight asymmetry of funnel plot suggests publication bias, which may distort findings in the direction of positive effects, a concern corroborated by the review's prioritization of peer‐reviewed studies, which may limit the inclusion of gray literature. However, this asymmetry could also be explained by genuine variance in populations and designs, such as a smaller number of group studies contributing to outliers. The substantial between‐study variance estimate (Tau^2^) reflects considerable differences among studies, attributable to both treatment‐related factors (e.g. U‐VBF protocols) and subject‐related factors (e.g. age), as discussed above. Taken together, these results concur with Sugden et al. (2019)’s positive outcomes; however, the present meta‐analysis quantifies this effect amid notable variability.

### 4.3 Strengths, Limitations and Future Directions

This review has several strengths that add to its contribution to the U‐VBF literature. The fact that it includes 35 studies with a greater number of participants, for example, provides a wider evidence base than the earlier review by Sugden et al. (2019), while the meta‐analysis based on 16 of these studies gives a quantitative grounding to the measurement of effectiveness. The incorporation of a variety of study designs represents a methodological improvement over the previous review, thereby improving result reliability.

However, several limitations temper these strengths. The pooled effect size may be subject to flawed or overestimated due to calculation approximations in some studies, such as lacking full participant SDs (requiring use of available reported data for d2 estimation) or missing SEs (leading to weighted summarization via proxies such as estimated number of trials to approximate participant‐level SEs before aggregating to study‐level d2), warranting caution in interpretation as these methods may introduce imprecision. Additionally, while the meta‐analysis covers nearly half the studies (16/35) and is representative of SCED‐dominant evidence, it primarily reflects single‐case designs and excludes most group studies, potentially underrepresenting more robust designs and biasing the pooled effect toward SCED‐specific positives. The focus on SSD in children and adolescents with exclusion of studies with mixed adult‐child cohorts may result in incomplete representation of relevant paediatric evidence and potentially neglect valuable insights from adult U‐VBF applications that might inform paediatric approaches. In addition, the reliance on peer‐reviewed publication reinforces possible publication bias, as suggested by the funnel plot asymmetry, which may limit the inclusion of negative or null results from unpublished studies and inflate the pooled effect size. Finally, the predominance of English‐speaking participants, consistent with the findings of Sugden et al. (2019), restricts cross‐linguistic generalizability and hinders applicability to non‐English phonologies. This limitation is not confined to phonology and phonetics but also extends to broader social and healthcare contexts, such as varying speech‐language therapy models in non‐Western settings.

Clinically, the real‐time visualization provided by U‐VBF supports therapists in providing specific feedback and complements client self‐correction. Building on these mechanisms, the enhanced reporting of treatment intensity facilitates personalized U‐VBF protocols, including dose or dose frequency titration for particular SSD subtype (e.g. higher doses for CAS than residual speech errors). Future research should follow up on the representation of RCTs within the 35 studies to increase generalizability beyond the prevalent single‐case designs, standardize reporting of participant variables and treatment intensity to detect differential responses, and investigate U‐VBF effectiveness in non‐English‐speaking populations and non‐Western contexts to increase global applicability, possibly using the effect size range to inform power calculations for future trials. To enable stronger future meta‐analyses and cross‐study comparisons, improved standardization of reporting—such as consistent documentation of intensity metrics and participant‐level data—is essential, as preliminary reviews highlight small but significant links between treatment intensity and efficacy while noting ongoing inconsistencies (Hitchcock et al. 2019).

## 5 Conclusion

This systematic review and meta‐analysis of U‐VBF for children and adolescents with SSD, updating Sugden et al. (2019), provides evidence of significant speech accuracy improvements despite substantial heterogeneity and wide variability in effect sizes. The inclusion of RCTs and better reporting of treatment intensity suggest methodological improvement, in addition to the widened evidence base with 192 unique participants, although generalizability is limited by publication bias, the predominance of English‐speaking participants and the exclusion of mixed‐age studies. Nonetheless, the large pooled effect and enhanced intensity documentation provide a robust foundation for clinical implementation. U‐VBF provides real‐time tongue visualization that aids clients in self‐correction and therapists in providing targeted feedback, as seen in the protocols described in the included studies. These findings support individualized interventions, potentially transforming SSD therapy by addressing persistent errors more effectively than traditional methods alone. To extend these insights, future treatment studies should prioritize RCTs and investigate non‐English populations, to strengthen the evidence base of U‐VBF for the optimization of therapy in children with SSD. Future reviews should incorporate gray literature to mitigate bias.

## Conflicts of Interest

The authors declare no conflicts of interest.

## Supporting information




**Supporting File**: jlcd70209‐supp‐0001‐Appendices.docx

## Data Availability

Data sharing is not applicable to this article as no datasets were generated or analysed during the current study.
