# Supplementary material for: The Effectiveness of Ultrasound Visual Biofeedback in Articulation Therapy for Children and Adolescents With Speech Sound Disorders: A Systematic Review and Meta‐Analysis
Source: Int J Lang Commun Disord. 2026 Feb 18;61(2):e70209. doi: 10.1111/1460-6984.70209 (PMC12914769; doi:10.1111/1460-6984.70209)
Supplement: Supplementary file 1 — Supporting File: jlcd70209‐supp‐0001‐Appendices.docx [file JLCD-61-0-s001.docx]

**SUPPLEMENTARY MATERIAL**

**Table S1**

*Comprehensive search strategy for systematic review*

| Database | Last date of search | Search string and filters |
| --- | --- | --- |
| Pubmed | July 1, 2025 | - (”ultrasonography”[MeSH]) - AND (”speech therapy”[MeSH] OR “speech-language pathology”[MeSH]) - AND (”speech disorders”[MeSH] OR “articulation disorders”[MeSH] OR “phonological disorders”[MeSH]) - AND (“child”[MeSH] OR “adolescent”[MeSH]) - Filters: Humans[Filter], Child: birth18 years[Filter], Date 2000/1/1 to 2025/7/1[pdat] |
| Web of Science | July 1, 2025 | - TS=(Ultrasound* OR sonography* OR sonogram* OR ultrasonography*) - AND TS=(speech therapy* OR speech pathology* OR ”speech language pathology”) - AND TS=(adolescent* OR youth OR child* OR teenager) - AND TS=(speech disorder*) - Filters: None |
| Linguistics and Language Behavior Abstracts | July 1, 2025 | - (ultrasound OR sonography OR sonogram OR ultrasonography) - AND (speech therapy OR speech pathology OR ”speech language pathology”) - AND (children OR adolescents OR youth OR child OR teenager) - AND (speech disorders) - Filters: Date 1/1/2000 to 07/01/2025 |

**Table S1 (continued)**

| Database | Last date of search | Search string and filters |
| --- | --- | --- |
| Cochrane library | July 1, 2025 | - (Ultrasound* OR sonography* OR sonogram* OR ultrasonography*) in All Text - AND (speech therapy* OR speech pathology* OR “speech language pathology*”) in All Text - AND (adolescent* OR youth OR child* OR teenager) in All Text - AND (Speech disorder) in All Text - Filters: None |
| CINAHL Plus | July 1, 2025 | - (Ultrasound* OR sonography* OR sonogram* OR ultrasonography* OR MH “Ultrasonography”) - AND (speech therapy* OR speech pathology* OR “speech language pathology*” OR MH “Speech Therapy”) - AND (adolescent* OR youth* OR child* OR teenager* OR MH ”Child” OR MH “Adolescent”) - AND (Speech disorder OR MH “Speech Disorders”) - Filters: Date 1/1/2000 to 07/01/2025 |

*Note*. MeSH = Medical Subject Headings. TS = Topic Search, searches title, abstract, author keywords, and Keywords Plus®. MH = Searches the exact CINAHL Plus Subject Heading, searching both major and minor headings

**Table S2**

*Participants and treatment details*

| **Study** | **Participants** | **Treatment details** | |  |
| --- | --- | --- | --- | --- |
|  | Description | Personnel, Conditions | Session Component | |
| Bernhardt et al., 2003 | *SSD:* not specified *Comorbidity:* hearing loss, hypernasality  *Spoken language:* English + Tagalog/Portuguese/Cantonese  *Prior therapy:* yes | SLP  *Conditions* - 3 sessions of U-VBF followed by 6 sessions of EPG or vice versa. Combined use for the last 5 sessions. | - SLP demonstrated treatment targets, and participants practised with progression of linguistic complexity, alongside the use of ultrasound or EPG equipment. | |
| Bernhardt et al., 2005 | *SSD:* not specified *Comorbidity:* hearing loss *Prior therapy:* yes  *Prior study:* yes (Bernhardt et al., 2003) | SLP | *First 9 sessions:* - 2 participants spent 6 sessions solely with EPG, and 3 sessions solely with ultrasound. - 2 participants spent 6 sessions solely with ultrasound, and 3 solely with EPG. *Final 5 sessions:* - Participants alternated between ultrasound and EPG. Targets progressed in linguistic complexity. | |
| Adler-Bock et al., 2007 | *SSD:* not specified  *Comorbidity:* none *Spoken language:* Canadian English *Prior therapy:* yes | SLP | 1. Awareness of components of /r/: Oriented to the ultrasound screen and tongue images and learnt articulatory requirements of /r/ production. 2. Production practice: Practised with modelling. Treatment targets progressed in linguistic complexity and from separate silent tongue gestures to voiced /r/. | |

**Table S2 (continued)**

| **Study** | **Participants** | **Treatment details** | |  |
| --- | --- | --- | --- | --- |
|  | Description | Personnel, Conditions | Session Component | |
| Bacsfalvi et al., 2007 | *SSD:* not specified *Comorbidity:* hearing loss  *Spoken language:* Western Canadian English (second language)  *Prior therapy:* yes *Prior study:* yes (Bernhardt et al., 2003) | Certified SLP | - Awareness Component: Phonetic instruction about the vowel quadrilateral and the tense-lax vowel distinction, with demonstrations using ultrasound or EPG images. - Vowel Practice: Vowels were practised in progression of linguistic complexity. | |
| Bernhardt et al, 2008 | *SSD:* residual speech impairment *Comorbidity:* minor oral deviation (n=10) Spoken language: not reported  *Prior therapy:* yes | SLP | *No U-VBF treatment phase:* - Details of session flow not mentioned *1–3 session U-VBF consultation:* - Training materials, including a DVD and manual, were provided to local SLPs, explaining ultrasound functionality and demonstrating tongue movements. - 2 northern communities received more ultrasound consultation: each child received 2–3 hours of ultrasound treatment (in three to four sessions) over 2 days. - The south-central communities received one session of ultrasound treatment per child in a half-day period.  *Second phase of no U-VBF treatment:* - Details of session flow not mentioned | |

**Table S2 (continued)**

| **Study** | **Participants** | **Treatment details** | |  |
| --- | --- | --- | --- | --- |
|  | Description | Personnel, Conditions | Session Component | |
| Modha et al., 2008 | *SSD:* not acquired /r/ naturally *Comorbidity:* restrictive frenulum *Spoken language:* Canadian English *Prior therapy:* yes | SLP  *Conditions* - with or without U-VBF. | - Across conditions, practice included imitative and spontaneous production. - Treatment targets progressed in linguistic complexity. - For no U-VBF - also included verbal descriptions of tongue placement and visual feedback with a mirror.  - For U-VBF - the subject and the SLPs took turns using the transducer. Treatment targets progressed in linguistic complexity and from separate silent tongue gestures to voiced /r/. | |
| Bacsfalvi, 2010 | *SSD:* not specified  *Comorbidity:* hearing loss *Spoken language:* English/Cantonese/sign language,  *Prior therapy:* yes, *Prior study:* yes (n=1, with U-VBF) | SLP | - Demonstration: Author demonstrated lingual components of /r/ (i.e., tongue root retraction and tongue tip retroflexion), allowing students to choose their preferred method. - Practice: Students progressed from lingual components to voicing, incorporating /r/ into syllables and words based on personal goals and contexts. | |

**Table S2 (continued)**

| **Study** | **Participants** | **Treatment details** | |  |
| --- | --- | --- | --- | --- |
|  | Description | Personnel, Conditions | Session Component | |
| Lipetz & Bernhardt, 2013 | *SSD:* residual frontal lisp *Comorbidity:* autism spectrum disorder *Spoken language:* English *Prior therapy:* yes | SLP student and clinician | *Phase 1 - General awareness of articulatory settings (AS):* - Direct education involved diagrams and models of vocal anatomy. - Voice training included exercises for jaw relaxation, postural demonstrations, and vocal stretches. - Techniques like the semi-occluded vocal tract were used to stretch vocal folds. - The participant practised changing AS while vocalizing vowels and reflecting on ultrasound images for specific sounds. *Phase 2 - Target phoneme practice:* - Phonemes were introduced in progression of linguistic complexity. - Once the participant was able to approximate the phoneme acoustic spectrographic feedback was applied to show the participant how to self-monitor his productions. | |
| Preston et al., 2013 | *SSD:* childhood apraxia of speech *Comorbidity:* none *Spoken language:* not reported, Prior therapy: yes | SLP or a graduate student supervised by SLP | 1. U-VBF (15min): Production training by utilizing a range of cues and strategies with U-VBF. 2. Tabletop Activities (8-10min): Used traditional speech sound training approaches such as drill and drill-play activities with speech sound training techniques like modelling, imitation, and phonetic cues related to articulatory positions and movements, and self-monitoring practice. | |

**Table S2 (continued)**

| **Study** | **Participants** | **Treatment details** | |  |
| --- | --- | --- | --- | --- |
|  | Description | Personnel, Conditions | Session Component | |
| Byun et al., 2014 - Study 1 | *SSD:* persistent rhotic errors *Comorbidity:* none *Spoken language:* monolingual English Prior therapy: yes (n=3) | Certified SLP  *Conditions* - 8 hr of high-frequency U-VBF (89% of trials) vs 8 hr of lower-frequency U-VBF (44% of trials) | *Instructional sessions (2 sessions):*  - Introduce sagittal targets with line drawings of major lingual constrictions and ultrasound images of bunched rhotic.  *Treatment sessions (14 sessions):*  1. Pre-practice: Production training by utilizing cues and strategies with discussions of tongue anatomy and articulatory requirements. 2. Treatment trials: Blocked practice of 5 trials (total: 30 trials of syllabic /ɝ/, followed by 10 trials each of syllables /aɹ/, /ai/, and /au/). | |
| Byun et al., 2014 - Study 2 | *SSD:* persistent rhotic errors *Comorbidity:* none *Spoken language:* monolingual English *Prior therapy:* yes | Certified SLP | *Instructional sessions (3 sessions):*  - Introduce sagittal targets with line drawings of major lingual constrictions, ultrasound images of bunched rhotic and magnetic resonance images of various rhotic tongue shapes.  *Treatment sessions (14 sessions):*  1. Pre-practice: Production training by utilizing cues and strategies with discussions of tongue anatomy and articulatory requirements. 2. Treatment trials: Blocked practice of 5 trials (total: 30 trials of syllabic /ɝ/, followed by 10 trials each of syllables /aɹ/, /ai/, and /au/). | |

**Table S2 (continued)**

| **Study** | **Participants** | **Treatment details** | |  |
| --- | --- | --- | --- | --- |
|  | Description | Personnel, Conditions | Session Component | |
| Cleland & Scobbie, 2015 | *SSD:* persistent primary SSD *Comorbidity:* none *Spoken language:* monolingual English *Prior therapy:* yes | SLP | 1. U-VBF (30min): Used pre-recorded videos of ultrasound as target movements and a visual articulatory model. Subjects progressed in linguistic complexity upon achieving 80% accuracy of the linguistic level. 2. Traditional tabletop activities for generalisation (30min): Progression to productions without U-VBF. | |
| Hitchcock & Byun, 2015 | *SSD:* persistent rhotic errors *Comorbidity:* none *Spoken language:* monolingual English *Prior therapy:* yes *Prior study:* yes (Byun et al., 2014) | SLP | 1. Free Play (5min): Practised /r/ targets using U-VBF in an unstructured environment, in which the clinician provided guidance and cues tailored to the session's focus. 2. Treatment (25-40min): Practice progressed in linguistic complexity, guided by CPF. Breaks were incorporated after every 10 trials. | |
| Lee et al, 2015 | *SSD:* developmental articulation disorder *Comorbidity:* developmental reading disorder, mixed expressive-receptive language disorder, auditory processing disorders *Spoken language:* monolingual English *Prior therapy:* yes | SLP (supervised by Certified SLP) | - In sessions 1-4, the subject was asked to produce /ɝ/ and watch his real-time tongue movement, while clinician demonstrated and produced /ʌ/ and /ɝ/. Productions of 2 different gestures of /r/ (i.e., retroflex vs. bunched) was attempted. Chocolate paste was introduced to help the patient practice curling the tongue tip back. - In sessions 5-6, produce subject was asked to produce /ʌ/ and /ɝ/ alternatively and contrastively. - In sessions 7-12, the subject's gestures were monitored by U-VBF. Then, tabletop activities without U-VBF were used for generalization. | |

**Table S2 (continued)**

| **Study** | **Participants** | **Treatment details** | |  |
| --- | --- | --- | --- | --- |
|  | Description | Personnel, Conditions | Session Component | |
| Bressmann et al., 2016 | *SSD:* unresolved /r/ articulation error *Comorbidity:* none *Spoken language:* Canadian English *Prior therapy:* not reported | SLP  *Conditions* - with or without U-VBF. | - Therapy materials were based on the "Wizard of R’s" collection, focusing on the target sound in various positions (initial, final, middle) and consonant clusters. - U-VBF (10-min): Instructions were provided to produce the retroflex variety of the /ɹ/ sound. | |
| Heng et al., 2016 | *SSD:* primary speech impairments involving /k/ and /g/ *Comorbidity:* none *Spoken language:* English  *Prior therapy:* yes | Researcher | 1. Pre-practice: Production training by utilizing a range of cues and strategies with U-VBF, aiming to achieve 3 correct productions of the target before advancing.  2. Practice: Required to produce the targets without U-VBF. 3-min breaks were incorporated after every 25 trials. | |
| Preston et al., 2016a | *SSD:* childhood apraxia of speech *Comorbidity:* none *Spoken language:* English *Prior therapy:* yes | Certified SLP | *- Production Training was divided into four 12-minute periods (A, B, C, and D). Periods A and C included ultrasound feedback, while B and D did not, to encourage generalization.* 1. Auditory Perception Training (6-10min): 50 trials of auditory perception training by judging recordings of correct and incorrect productions. Each child was exposed to at least 100 different tokens of each sound in each word position. 2. Production Training (48min):  - Pre-practice: Unstructured production training by utilizing a range of cues and strategies with U-VBF, aiming to achieve 12 correct productions of target sounds before advancing. - Structured Practice: By using speech motor chaining, practice progressed in linguistic complexity. | |

**Table S2 (continued)**

| **Study** | **Participants** | **Treatment details** | |  |
| --- | --- | --- | --- | --- |
|  | Description | Personnel, Conditions | Session Component | |
| Preston et al., 2016b | *SSD:* childhood apraxia of speech *Comorbidity:* none *Spoken language:* monolingual English *Prior therapy:* yes | Certified SLP  *Conditions* - with or without prosodic cueing | *- Each session was divided into four 13-minute time periods. Periods A and C included practise with the ultrasound; Periods B and D did not. - Prosodic cueing: pairing the target utterance with one of three punctuation cues (question mark, exclamation point, and period) was included in one randomly-selected phase.* 1. Pre-practice: Unstructured production training by utilizing a range of cues and strategies, aiming to achieve 6 correct productions of target sound /ɹ/ in each of two phonetic contexts before advancing. 2. Structured practice: Blocked practice of 6 trials, with progression in linguistic complexity. | |
| Roxburgh et al., 2016 | *SSD:* not reported Comorbidity: submucous cleft palate *Spoken language:* not reported Prior therapy: yes | SLP | 1. U-VBF: - Used pre-recorded videos of ultrasound as target movements and a visual articulatory model. - Production practice included basic articulation hierarchy or motor-based approach. Subjects progressed in linguistic complexity upon achieving 80% accuracy of the linguistic level. 2. Traditional tabletop activities for generalisation: Progression to productions without U-VBF. | |

**Table S2 (continued)**

| **Study** | **Participants** | **Treatment details** | |  |
| --- | --- | --- | --- | --- |
|  | Description | Personnel, Conditions | Session Component | |
| Sjolie et al., 2016 | *SSD:* primary SSD  *Comorbidity:* no CAS/ developmental disabilities/ syndrome *Spoken language:* monolingual American English *Prior therapy:* yes | SLP (supervised by Certified SLP)  *Conditions* - with or without U-VBF | 1. Pre-practice (4-6min): Unstructured production training of target sounds by utilizing a range of cues and strategies, aiming to achieve six correct renditions of two targets before advancing. 2. Structured Practice: By using speech motor chaining, practice progressed in linguistic complexity. 3. Random Practice (6min): Random practice of the highest linguistic level achieved. | |
| Preston et al., 2017a | *SSD:* Residual speech errors (RSEs)  *Comorbidity:* no developmental disabilities or cognitive delays *Spoken language:* rhotic American English *Prior therapy:* yes (n=8) | SLP  *Conditions* - PML + U-VBF then U-VBF + PML and vice versa | 1. Pre-practice: Unstructured production training of target sounds by utilizing a range of cues and strategies, aiming to achieve six correct renditions of two targets before advancing. 2. Structured Practice: By using speech motor chaining, practice progressed in linguistic complexity. | |

**Table S2 (continued)**

| **Study** | **Participants** | **Treatment details** | |  |
| --- | --- | --- | --- | --- |
|  | Description | Personnel, Conditions | Session Component | |
| Preston et al., 2017b | *SSD:* childhood apraxia of speech *Comorbidity:* none Spoken language: not reported *Prior therapy:* not reported | Certified SLP  *Conditions* - prosodic or non-prosodic (PROS and No-PROS). | 1. Auditory Perception Training (8min): 50 trials of auditory perception training by judging recordings of correct and incorrect productions. 2. Production Practice (20min):  - Pre-practice: Unstructured production training by utilizing verbal and visual instructions, aiming to achieve 3 correct productions of each of the 4 syllable variants targets before advancing. (PROS: prosodic variations were modelled by the SLP for each production; No-PROS: without intentional prosodic manipulation, using a neutral tone) - Structured practice: Blocked practice of 6 trials, with progression in linguistic complexity. 3. Repeat auditory perception training and production practice for second target | |
| Preston et al., 2018 | *SSD:* RSEs *Comorbidity:* none *Spoken language:* rhotic dialects of North American English *Prior therapy:* yes (n=10) | SLP  *Conditions* - 8 hr of high-frequency U-VBF (89% of trials) vs 8 hr of lower-frequency U-VBF (44% of trials) | 1. Pre-practice (5min): Unstructured production training by utilizing traditional cues and strategies with U-VBF with discussions of tongue anatomy and articulatory requirements. 2. Structured Practice (162 practice attempts/45min): Practice progressed in linguistic complexity (blocks of six trials), guided by CPF. | |

**Table S2 (continued)**

| **Study** | **Participants** | **Treatment details** | |  |
| --- | --- | --- | --- | --- |
|  | Description | Personnel, Conditions | Session Component | |
| Cleland et al., 2019 | *SSD:* mixed – inconsistent phonological disorder (n=1), childhood apraxia of speech (n=4), phonological delay (n=6), articulation disorder (n=3), phonological disorder (n=1)  *Comorbidity:* developmental language disorder (n=2), Autism Spectrum Disorder (ASD) (n=2), Attention Deficit Disorder/ ASD (n=1)  *Spoken language:* English  *Prior therapy:* yes | SLP | 1. U-VBF (30min): Used pre-recorded videos of ultrasound as target movements and a visual articulatory model. Subjects progressed in linguistic complexity upon achieving 80% accuracy of the linguistic level. 2. Traditional tabletop activities for generalisation (30min): Progression to productions (drill production of around 100 words with target segment at each level) without U-VBF. | |
| Preston et al., 2019 | *SSD:* RSEs *Comorbidity:* no development disability *Spoken language:* rhotic dialects of North American English *Prior therapy:* not reported | SLP  *Conditions* - With or without U-VBF | 1. Pre-practice (5min): Unstructured production training by utilizing traditional cues and strategies after a discussion of tongue anatomy and articulatory requirements. (U-VBF: first treatment session featured an initial overview of how to interpret ultrasound images) 2. Structured Practice (162 trials/45min): Practice progressed in linguistic complexity (blocks of six trials), using the Challenge-R software. | |

**Table S2 (continued)**

| **Study** | **Participants** | **Treatment details** | |  |
| --- | --- | --- | --- | --- |
|  | Description | Personnel, Conditions | Session Component | |
| Preston et al., 2020 | *SSD:* /r/ distortion *Comorbidity:* none  *Spoken language:* not reported *Prior therapy:* yes (78.5%) | Certified SLP  *Conditions* - with or without auditory perceptual training (P+U-VBF and U-VBF) | 1. Pre-practice (6-8min): Unstructured production training by utilizing a range of cues and strategies with U-VBF, aimed at achieving correct productions of target sounds. (P+U-VBF condition: additional perceptual training with 50 audio judgments) 2. Structured Practice (162 trials/45min): Practice progressed in linguistic complexity, guided by CPP. (U-VBF: Clinician-provided feedback only; P+U-VBF: Added self-rating on 50% of trials) | |
| Benway et al., 2021 | *SSD:* RSEs *Comorbidity:* no neurobehavioral disorder or childhood apraxia of speech *Spoken language:* monolingual American English *Prior therapy:* not reported | SLP | 1. Pre-practice (3 correct productions/15min): Production training by utilizing traditional cues and strategies with U-VBF. 2. Structured Practice (200 trials/30min): Blocked practice of 10 trials, guided by a standardized using customized, open-source software for stimulus presentation. | |
| Cleland & Scobbie, 2021 | *SSD:* inconsistent speech disorder (n=1), childhood apraxia of speech (n=1) and phonological delay (n=3) *Comorbidity:* none  *Prior therapy:* yes  *Prior study:* yes (Cleland et al., 2019) | SLP | 1. U-VBF: Used pre-recorded videos of ultrasound as target movements and a visual articulatory model. Subjects progressed in linguistic complexity upon achieving 80% accuracy of the linguistic level. 2. Traditional tabletop activities for generalisation: Progression to productions (drill production of around 100 words with target segment at each level) without U-VBF. | |

**Table S2 (continued)**

| **Study** | **Participants** | **Treatment details** | |  |
| --- | --- | --- | --- | --- |
|  | Description | Personnel, Conditions | Session Component | |
| Gibson & Lee, 2021 | *SSD:* not specified,  *Comorbidity:* hearing loss, *Spoken language:* English,  *Prior therapy:* yes | SLP (supervised by Certified SLP) | *Pre-intervention Training (2 sessions):*  1. Familiarization with the ultrasound equipment, by viewing tongue shapes on ultrasound monitor. *Treatment Sessions:* 1. Ultrasound Phase: Unstructured production training by utilizing a range of cues and strategies with real-time U-VBF with a discussion of tongue anatomy and articulatory requirements. 2. Tabletop Phase: Various games and drill activities were integrated, where children produced target consonants in isolation or within syllables. | |
| Raaz et al., 2021 | *SSD:* RSEs *Comorbidity:* none *Prior therapy:* not reported *Prior study:* yes (Preston et al., 2020) | SLP | 1. Pre-practice: 50 items of speech perception training using the Speech Assessment and Interactive Learning System (SAILS), by judging recordings of correct and incorrect productions (recorded productions of /ɹ/). 2. Structured Practice (45min): Practice of 162 trials progressed in linguistic complexity, guided by CPP. | |

**Table S2 (continued)**

| **Study** | **Participants** | **Treatment details** | |  |
| --- | --- | --- | --- | --- |
|  | Description | Personnel, Conditions | Session Component | |
| McAllister et al., 2022 | *SSD:* RSEs *Comorbidity:* none *Spoken language:* rhotic dialects of American English *Prior therapy:* not reported | SLP | *Phase I (1.5hr/session):*  1. Pre-practice (All target syllables produced correctly 3 times/50min): Unstructured production training by utilizing a range of cues and strategies. 2. Structured practice: Produced 64 trials guided by CPP. *Phase II-a (1.5hr/session):*  1. Pre-practice (24 correct productions/50min): Same as phase I 2. Structured practice (30min): Produced 216 trials, in blocks of six trials, with real-time U-VBF. *Phase II-b (45min-1hr/session):*  1. Pre-practice (24 correct productions/15min): Same as phase I 2. Structured practice: Same as Phase II-a, but guided by CPP. | |

**Table S2 (continued)**

| **Study** | **Participants** | **Treatment details** | |  |
| --- | --- | --- | --- | --- |
|  | Description | Personnel, Conditions | Session Component | |
| McCabe et al., 2023 | *SSD:* childhood apraxia of speech *Comorbidity:* none *Spoken language:* English *Prior therapy:* yes *Prior study:* yes (not involving U-VBF) | SLP (supervised by SLP)  *Conditions* - U-VBF with motor chaining procedures or ReST | 1. Pre-practice:  - ReST (<20min) - production training concentrating on accurate production, stress patterns, and smooth/connected productions; aiming to achieve five accurate productions of any target word(s) before advancing. - U-VBF - production training by utilizing modeling and verbal instructions with U-VBF, aiming to achieve six correct productions for each sound sequence before advancing. 2. Practice: Practice progressed in linguistic complexity. (U-VBF group used speech motor chaining procedures divided into 12-min therapy periods; while ReST group followed the procedures in the ReST Manual.) | |
| Spencer et al., 2023 | *SSD:* RSEs *Comorbidity:* no neurologically based speech disorder *Spoken language:* not reported *Prior therapy:* not reported | Not reported | - Practised up to 162 trials, progressing in linguistic complexity, guided by the Challenge Point software. | |

**Table S2 (continued)**

| **Study** | **Participants** | **Treatment details** | |  |
| --- | --- | --- | --- | --- |
|  | Description | Personnel, Conditions | Session Component | |
| Preston et al., 2024 | *SSD:* childhood apraxia of speech *Comorbidity:* not reported *Spoken language:* American English *Prior therapy:* yes | SLP or Clinical Fellow (supervised by SLP)  *Conditions* - Distributed + Ultrasound, Intensive + Ultrasound, Distributed + No Ultrasound, and Intensive + No Ultrasound | 1. Phonological Awareness Training (5-10min): Produced 10 trials of targets, demonstrating sound changes based on clinician instructions. 2. Pre-practice (3 correct productions of each of the 4 syllable variants/24min): Unstructured production training by utilizing a range of cues and strategies with U-VBF (for U-VBF condition), or static visual references (for no U-VBF condition). 3. Structured Practice (remaining of the 24min of (2)): By using speech motor chaining, practice progressed in linguistic complexity. 4. Random Practice (5-10min): Random practice of 30 trials (mean) the highest linguistic level achieved, with prosodic variations introduced. | |
| Hashemi Hosseinabad & Xing, 2024 | *SSD:* persistent speech errors *Comorbidity:* cleft palate, auditory processing disorder, ASD, delayed language development, language disorder. Syndrome - 22q deletion (n=2), Turner syndrome (n=1) *Spoken language:* English *Prior therapy:* yes | Certified SLP | 1. U-VBF (30min): Used pre-recorded videos of ultrasound as target movements and a visual articulatory model. The children progressed to the next level of complexity as soon as they were able to achieve the previous level at 80%. 2. Traditional tabletop activities for generalisation (15min): Progression to productions without U-VBF. | |

*Note.* SSD = Speech Sound Disorder, SLP = Speech Language Pathologist, U-VBF = Ultrasound-Visual Biofeedback, EPG = Electropalatography, CPF = Challenge Point Framework, PML = Principle of Motor Learning, CPP = Challenge Point Program, ReST = Rapid Sayllable Transitions, RSE = Residual speech errors

**Table S3**

*Extracted Data and d2 Effect Size Derivations for Meta-Analysis Studies*

| **Study** | **Pre- and Post- Intervention Means and SDs  (per participant)** | **Data Points per phase (Pre/ Post)** | **Individual-level d2 and SEs** | **Notes on approximation** | **Study level d2** | **Study level SE** | **n** |
| --- | --- | --- | --- | --- | --- | --- | --- |
| Bacsfalvi, 2010 | Parker  Pre: M=36.18, SD=29.16  Post: M=68.51, SD=22.43  Pearl  Pre: M=20.89, SD=10.69  Post: M=21.91, SD=10.23  Petra  Pre: M=30.43, SD=11.50  Post: M=37.49, SD=13.23 | Pre n=3,  Post n=3 (all) | *Parker: d2=1.24, SE=1.03*  *Pearl: d2=0.10, SE=0.70*  *Petra: d2=0.57, SE=0.85* | - | *0.49* | *0.48* | 3 |
| Lipetz & Bernhardt, 2013 | Pre: M=22.21, SD=27.64  Post: M=83.54, SD=17.63 | Pre n=7,  Post n=7 | *d2=2.65, SE=1.25* | - | *2.65* | *1.25* | 1 |

**Table S3** (continued)

| **Study** | **Pre- and Post- Intervention Means and SDs  (per participant)** | **Data Points per phase (Pre/ Post)** | **Individual-level d2 and SEs** | **Notes on approximation** | **Study level d2** | **Study level SE** | **n** |
| --- | --- | --- | --- | --- | --- | --- | --- |
| Preston et al., 2013 | U002  Pre: M=0.153, SD=0.122  Post: M=0.630, SD=0.092  U005  Pre: M=0.263, SD=0.174  Post: M=0.783, SD=0.138  U007  Pre: M=0.124, SD=0.079  Post: M=0.436, SD=0.108  U008  Pre: M=0.312, SD=0.199  Post: M=0.896, SD=0.075  U009  Pre: M=0.302, SD=0.169  Post: M=0.885, SD=0.102  U012  Pre: M=0.283, SD=0.163  Post: M=0.905, SD=0.063 | U002  Pre n=3,  Post n=7  U005  Pre n=6,  Post n=6  U007  Pre n=5,  Post n=5  U008  Pre n=5,  Post n=5  U009  Pre n=6,  Post n=6  U012  Pre n=6,  Post n=6 | *U002: d2=4.40, SE=1.63*  *U005: d2=3.31, SE=1.39*  *U007: d2=3.31, SE=1.41*  *U008: d2=3.87, SE=1.50*  *U009: d2=4.18, SE=1.54*  *U012: d2=5.04, SE=1.67* | - | *3.94* | *0.62* | 6 |

**Table S3** (continued)

| **Study** | **Pre- and Post- Intervention Means and SDs  (per participant)** | | **Data Points per phase (Pre/ Post)** | **Individual-level d2 and SEs** | **Notes on approximation** | **Study level d2** | **Study level SE** | **n** |  |
| --- | --- | --- | --- | --- | --- | --- | --- | --- | --- |
| Byun et al., 2014 - Study 1 | | Not reported | | | *Neville: d2=-1.05*  *Gabby: d2=1.55*  *Mina: d2=2.3*  *David: d2=0.15* | Individual d2 = average of the vocalic and consonantal variants  Number of trials = 64 | *0.45* | *0.1* | 4 |
| Byun et al., 2014 - Study 2 | | Not reported | | | *Philip: d2=3*  *Lilianne: d2=10.35*  *Autumn: d2=12.35*  *Jordan: d2=3.6* | Individual d2 = average of the vocalic and consonantal variants  Number of trials = 64 | *4.06* | *0.23* | 4 |
| Heng et al., 2016 | | P1  Pre: M=0.011, SD=0.016  Post: M=0.177, SD=0.062 | Pre n=2,  Post n=2 | | *d2=3.66, SE=1.53* | P3 is not included as pre-and post-SD = 0 | *3.66* | *1.53* | 1 |

**Table S3** (continued)

| **Study** | **Pre- and Post- Intervention Means and SDs  (per participant)** | **Data Points per phase (Pre/ Post)** | **Individual-level d2 and SEs** | **Notes on approximation** | **Study level d2** | **Study level SE** | **N** |
| --- | --- | --- | --- | --- | --- | --- | --- |
| Preston et al., 2016b | 94  Pre: M=1.5, SD=2.12  Post: M=3, SD=1.41  97  Pre: M=4.5, SD=6.36  Post: M=12, SD=7.07  103  Pre: M=1, SD=1.41  Post: M=0.5, SD=0.707 | Pre n=2,  Post n=2  (all) | *94: d2=0.83, SE=0.96*  *97: d2=1.11, SE=1.03*  *103: d2=-0.45, SE=0.53* | - | *0.06* | *0.42* | 3 |
| Sjolie et al., 2016 | 1003  Pre: M=29.4, SD=15.6  Post: M=42.6, SD=18.2  1004  Pre: M=2.86, SD=7.56  Post: M=0, SD=0  1008  Pre: M=34.2, SD=19.8  Post: M=36.8, SD=21.2 | Pre n=10,  Post n=10  (all) | *1003: d2=0.78, SE=0.75*  *1004: d2=-0.54, SE=0.30*  *1008: d2=0.13, SE=0.49* | Subject 1010 is not included as pre-and post-SD = 0 | *-0.24* | *0.24* | 3 |

**Table S3** (continued)

| **Study** | **Pre- and Post- Intervention Means and SDs**  **(per participant)** | **Data Points per phase** **(Pre/ Post)** | **Individual-level d2 and SEs** | **Notes on approximation** | **Study level d2** | **Study level SE** | **N** |
| --- | --- | --- | --- | --- | --- | --- | --- |
| Preston et al., 2017a | Not reported |  | A: d2=20.59  B: d2=4.01  C: d2=1.38  D: d2=4.79  E: d2=1.32  F: d2=0.74  G：d2=1.34  H: d2=11.19  I: d2=2.44  J: d2=14.56  K: d2=-0.43  L：d2=0.15 | Number of trials = 53 | *1.13* | *0.08* | 12 |
| Preston et al., 2017b | Not reported |  | Danica: d2=36.65  Ethan: d2=8.71  Finn: d2=1.75  Greg: d2=1.42  Hannah: d2=1.51  Isaac: d2=-0.19 | Number of trials = 3 | *1.01* | *0.41* | 6 |

**Table S3** (continued)

| **Study** | **Pre- and Post- Intervention Means and SDs**  **(per participant)** | **Data Points per phase** **(Pre/ Post)** | **Individual-level d2 and SEs** | **Notes on approximation** | **Study level d2** | **Study level SE** | **N** |
| --- | --- | --- | --- | --- | --- | --- | --- |
| Preston et al., 2018 | Not reported |  | 125: d2=24.41  126: d2=5.56  127: d2=94.2  122：d2=1.4  113: d2=0.53  124：d2=10.2  119：d2=8.51  111：d2=-1.47  112: d2=6.89  114：d2=6.76  115: d2=1.73  128: d2=1 | Number of trials = 50 | *1.25* | *0.1* | 12 |
| Preston et al., 2019 | Not reported |  | 132: d2=21.34  139: d2=8.56  129: d2=-0.34  135: d2=8.25  140: d2=-1.15  142: d2=12.28  130: d2=7.96  134: d2=-12.52  131: d2=-7.83  136: d2=-6.25  141: d2=-0.56  143: d2=8.91 | Number of trials = 50 | *-0.26* | *0.11* | 12 |

**Table S3** (continued)

| **Study** | **Pre- and Post- Intervention Means and SDs**  **(per participant)** | **Data Points per phase** **(Pre/ Post)** | **Individual-level d2 and SEs** | **Notes on approximation** | **Study level d2** | **Study level SE** | **N** |
| --- | --- | --- | --- | --- | --- | --- | --- |
| Benway et al., 2021 | Not reported |  | 3101: d2=1.01  3102: d2=0.64  3104: d2=0  6102: d2=0.09  6103: d2=3.11  6104: d2=0  6108: d2=1.71 | Number of trials = 50 | *0.6* | *0.08* | 7 |
| Gibson & Lee, 2021 | Emily  Pre: M=0, SD=0  Post: M=0.67, SD=0.31  Anna  Pre: M=0, SD=0  Post: M=0.73, SD=0.12 | Pre n=3,  Post n=3  (all) | *Emily: d2=3.09, SE=1.41*  *Anna: d2=8.98, SE=2.22* | - | *4.78* | *1.19* | 2 |

**Table S3** (continued)

| **Study** | **Pre- and Post- Intervention Means and SDs**  **(per participant)** | **Data Points per phase** **(Pre/ Post)** | **Individual-level d2 and SEs** | **Notes on approximation** | **Study level d2** | **Study level SE** | **N** |
| --- | --- | --- | --- | --- | --- | --- | --- |
| Spencer et al., 2023 | Not reported |  | UC_1: d2=3.39  UC_2: d2= 12.42  UC_3: d2=-5.51  UC_4: d2=2.76  UC_5: d2=13.02  UC_10: d2=20.57  UC_11: d2=1.24  UC_12: d2=7.27  UC_14: d2=3.26  UC_15: d2=-3.24  UC_17: d2=14.31  UC_18: d2=0.02  UC_19: d2=6.22  UC_20: d2=-0.36  UC_21: d2=8.46  UC_22: d2=3.35 | Number of trials = 50 | *1.15* | *0.09* | 16 |
| Hashemi Hosseinabad & Xing, 2024 | Not reported |  | CLP01: d2=2.25  CLP03: d2=1.65  CLP05: d2=3.12  CLP08: d2=1.11  CLP09: d2=3.17 | Number of trials = 50 | *1.95* | *0.13* | 5 |

*Note.* d2=Busk & Serlin effect size; SE = Standard Error; M = Mean; SD = Standard Deviation.

Values in italics (e.g., individual-level d2, study-level d2 and SEs) represent calculations derived by the authors from extracted data;
Non-italicized values (e.g., pre/post means/SDs, individual-level d2) are reported in the original studies.
